# Differential Contribution of Subunit Interfaces to *α*9*α*10 Nicotinic Acetylcholine Receptor Function

**DOI:** 10.1124/mol.116.107482

**Published:** 2017-03

**Authors:** Juan Carlos Boffi, Irina Marcovich, JasKiran K. Gill-Thind, Jeremías Corradi, Toby Collins, María Marcela Lipovsek, Marcelo Moglie, Paola V. Plazas, Patricio O. Craig, Neil S. Millar, Cecilia Bouzat, Ana Belén Elgoyhen

**Affiliations:** Instituto de Investigaciones en Ingeniería, Genética y Biología Molecular, Dr Héctor N Torres (J.C.B., I.M., M.M. L., M.M., P.V.P., A.B.E.), Instituto de Química Biológica (P.O.C.), and Instituto de Investigaciones Bioquímicas de Bahía Blanca (J.C., C.B), Consejo Nacional de Investigaciones Científicas y Técnicas, Buenos Aires, Argentina; Department of Neuroscience, Physiology and Pharmacology, University College London, United Kingdom (J.K.G.-T., T.C., N.S.M.); Departamento de Química Biológica Facultad de Ciencias Exactas y Naturales (P.O.C.), and Instituto de Farmacología, Facultad de Medicina (P.V.P., A.B.E.), Universidad de Buenos Aires, Buenos Aires, Argentina; and Departamento de Biología, Bioquímica y Farmacia, Universidad Nacional del Sur, Bahía Blanca, Argentina (J.C., C.B).

## Abstract

Nicotinic acetylcholine receptors can be assembled from either homomeric or heteromeric pentameric subunit combinations. At the interface of the extracellular domains of adjacent subunits lies the acetylcholine binding site, composed of a principal component provided by one subunit and a complementary component of the adjacent subunit. Compared with neuronal nicotinic acetylcholine cholinergic receptors (nAChRs) assembled from *α* and *β* subunits, the *α*9*α*10 receptor is an atypical member of the family. It is a heteromeric receptor composed only of *α* subunits. Whereas mammalian *α*9 subunits can form functional homomeric *α*9 receptors, *α*10 subunits do not generate functional channels when expressed heterologously. Hence, it has been proposed that *α*10 might serve as a structural subunit, much like a *β* subunit of heteromeric nAChRs, providing only complementary components to the agonist binding site. Here, we have made use of site-directed mutagenesis to examine the contribution of subunit interface domains to *α*9*α*10 receptors by a combination of electrophysiological and radioligand binding studies. Characterization of receptors containing Y190T mutations revealed unexpectedly that both *α*9 and *α*10 subunits equally contribute to the principal components of the *α*9*α*10 nAChR. In addition, we have shown that the introduction of a W55T mutation impairs receptor binding and function in the rat *α*9 subunit but not in the *α*10 subunit, indicating that the contribution of *α*9 and *α*10 subunits to complementary components of the ligand-binding site is nonequivalent. We conclude that this asymmetry, which is supported by molecular docking studies, results from adaptive amino acid changes acquired only during the evolution of mammalian *α*10 subunits.

## Introduction

Nicotinic acetylcholine (ACh) receptors (nAChRs) are members of the pentameric ligand-gated ion channel family (Nemecz et al., 2016). Seventeen nAChR subunits (*α*1–*α*10, *β*1–*β*4, *δ*, *γ*, and *ε*) have been identified in vertebrates (Nemecz et al., 2016), each of which has a large extracellular N-terminal region, four transmembrane helices (M1–M4), and an intracellular domain (Thompson et al., 2010). At the interface of the extracellular domains of adjacent subunits lies the ACh binding site, formed by six noncontiguous regions (loops A–F). Each binding site is composed of a principal component or (+) face provided by one subunit, which contributes three loops of highly conserved residues (loops A–C), and a complementary component (−) of the adjacent subunit, which contributes three loops (D–F) that have lower levels of sequence conservation between subunits (Brejc et al., 2001; Unwin, 2005; Dellisanti et al., 2007). Consequently, the components of the extracellular intersubunit binding sites are nonequivalent and their loops contribute differently to receptor function (Karlin, 2002).

nAChRs can be assembled from either homomeric or heteromeric subunit combinations (Millar and Gotti, 2009). Homomeric receptors, such as *α*7, have five equivalent ACh binding sites, each formed by the same principal and complementary components. ACh occupancy of one site is enough for activation of the homomeric human *α*7 nAChR, and also of a chimeric receptor containing the extracellular domain of *α*7 and the transmembrane domain of the serotonin type 3A (5-HT3A) receptor subunit (Rayes et al., 2009; Andersen et al., 2013). However, whereas occupancy of three nonconsecutive binding sites is required for maximal open-channel lifetime of the chimeric receptor, only one functional agonist binding site is required for maximal open-channel lifetime in *α*7.

In contrast to homomeric nAChRs, heteromeric receptors can have nonequivalent ACh binding sites provided by different subunit interfaces. For example, the *Torpedo* nAChR has two structurally different binding sites provided by the *α*(+)-*δ*(−) and *α*(+)-*γ*(−) subunit interfaces (Martinez et al., 2000), which bind agonists with different affinities (Blount and Merlie, 1989; Prince and Sine, 1999). According to the known stoichiometries of some neuronal nAChRs (Millar and Gotti, 2009), and by analogy to the muscle-type receptor, it was originally thought that heteromeric nAChRs have two agonist binding sites (Sine, 2002). In the case of neuronal nAChRs such as *α*4*β*2, *α* subunits were thought to only provide the (+) site to the binding interface, whereas *β* subunits were thought to provide the (−) site (Arias, 1997; Luetje and Patrick, 1991). However, it was subsequently shown that the composition of binding site interfaces is more complex. For example, the *α*4*β*2 receptor has two alternative stoichiometries, (*α*4)_2_(*β*2)_3_ and (*α*4)_3_(*β*2)_2_, leading to different binding site configurations and resulting in different functional and pharmacological properties (Carbone et al., 2009; Harpsøe et al., 2011; Mazzaferro et al., 2011); whereas (*α*4)_2_(*β*2)_3_ has two agonist binding sites provided by *α*(+)-*β*(−) interfaces, (*α*4)_3_(*β*2)_2_ has a third *α*(+)-*α*(−) binding interface (Hsiao et al., 2008; Mazzaferro et al., 2011).

The *α*9*α*10 receptor is an atypical member of the nAChR family. It is a heteromeric receptor composed only of *α* subunits (Elgoyhen et al., 1994, 2001; Sgard et al., 2002). Mammalian *α*9 subunits can form functional homomeric *α*9 receptors with an EC_50_ for ACh similar to that of the heteromeric *α*9*α*10 receptor (Elgoyhen et al., 1994, 2001). Hence, *α*9 subunits are capable of providing principal and complementary components to functional agonist binding sites. In contrast, rat and human *α*10 subunits do not lead to functional channels when expressed heterologously (Elgoyhen et al., 2001; Sgard et al., 2002). Consequently, it has been proposed that *α*10 might serve as a structural subunit, much like a *β* subunit of heteromeric receptors, providing only complementary components to the agonist binding site (Elgoyhen and Katz, 2012). A (*α*9)_2_(*α*10)_3_ stoichiometry has been determined for the rat recombinant receptor (Plazas et al., 2005), although expression of a 10-fold excess of *α*9 compared with *α*10 in *Xenopus* oocytes can lead to an additional receptor isoform with the stoichiometry (*α*9)_3_(*α*10)_2_ (Indurthi et al., 2014). However, the relative contribution of each subunit to the binding pockets of the heteromeric *α*9*α*10 receptor is unknown. By a combination of approaches (site-directed mutagenesis, expression studies, and molecular docking) we show that, contrary to previous assumptions, *α*10 subunits do contribute to the principal component of the binding site. Moreover, the contribution of *α*9 and *α*10 to the complementary component is nonequivalent. Our results demonstrate the versatility of nAChR subunits to generate diverse binding site interfaces with potentially different functional and/or pharmacological properties.

## Materials and Methods

### 

#### Expression of Recombinant Receptors in *Xenopus laevis* oocytes.

cDNAs encoding *Gallus gallus* (chick) or *Rattus norvegicus* (rat) *α*9 and *α*10 nAChR subunits were subcloned into a modified pGEMHE vector for expression studies in *Xenopus laevis* oocytes. Capped cRNAs were in vitro transcribed from linearized plasmid DNA templates using RiboMAX Large Scale RNA Production System (Promega, Madison, WI). Mutant subunits were produced using Quick Change XL II kit (Stratagene, La Jolla, CA). Amino acid sequences of rat and chicken *α*9, *α*10, and *Torpedo* *α*1 subunits were aligned using ClustalW (EMBL-EBI, Wellcome Genome Campus, Hinxton, Cambridgeshire). Residues were numbered according to the corresponding *Torpedo* *α*1 subunit mature protein (Karlin, 2002).

The maintenance of *Xenopus laevis* and the preparation and cRNA injection of stage V and VI oocytes have been described in detail elsewhere (Verbitsky et al., 2000). Typically, oocytes were injected with 50 nl of RNase-free water containing 0.01–1.0 ng of cRNA (at a 1:1 molar ratio when pairwise combined) and maintained in Barth’s solution at 18°C. Electrophysiological recordings were performed 2–6 days after cRNA injection under two-electrode voltage clamp with an Oocyte Clamp OC-725B or C amplifier (Warner Instruments Corp., Hamden, CT). Recordings were filtered at a corner frequency of 10 Hz using a 900BT Tunable Active Filter (Frequency Devices Inc., Ottawa, IL). Data acquisition was performed using a Patch Panel PP-50 LAB/1 interface (Warner Instruments Corp.) at a rate of 10 points per second. Both voltage and current electrodes were filled with 3 M KCl and had resistances of ∼1 MΩ. Data were analyzed using Clampfit from the pClamp 6.1 software (Molecular Devices, Sunnyvale, CA). During electrophysiological recordings, oocytes were continuously superfused (∼15 ml/min) with normal frog saline composed of: 115 mM NaCl, 2.5 mM KCl, 1.8 mM CaCl_2_, and 10 mM HEPES buffer, pH 7.2. ACh was added to the perfusion solution for application. Unless otherwise indicated, the membrane potential was clamped to −70 mV. To minimize activation of the endogenous Ca^2+^ sensitive chloride current (Elgoyhen et al., 2001), all experiments were performed in oocytes incubated with the Ca^2+^ chelator 1,2-bis(2-aminophenoxy)ethane-*N*,*N*,*N*′,*N*′-tetraacetic acid (acetoxymethyl ester) (100 *µ*M) for 3 hours before electrophysiological recordings.

Concentration-response curves were normalized to the maximal agonist response in each oocyte. The mean and S.E.M. values of the responses are represented. Agonist concentration-response curves were iteratively fitted, using Prism 5 software (GraphPad Software Inc., La Jolla, CA), with the equation: *I*/*I*_max_ = A^nH^/(A^nH^ + EC_50_^nH^), where *I* is the peak inward current evoked by the agonist at concentration A; *I*_max_ is the current evoked by the concentration of the agonist eliciting a maximal response; EC_50_ is the concentration of the agonist inducing a half-maximal current response; and nH is the Hill coefficient. Data were analyzed using Clampfit from the pClamp 6.1 software.

The effects of extracellular Ca^2+^ on the ionic currents through mutant *α*9*α*10 receptors were studied by measuring the amplitudes of the responses to an EC_50_ concentration of ACh upon varying the concentration of this cation from nominally 0 to 3 mM (Weisstaub et al., 2002). Amplitude values obtained at each Ca^2+^ concentration were normalized to that obtained in the same oocyte at a 1.8 mM. Values from different oocytes were averaged and expressed as the mean ± S.E.M.

#### Radioligand Binding.

Chimeric subunit cDNAs containing the extracellular N-terminal domain of the *α*9 or *α*10 subunit fused to the transmembrane and intracellular domain of the mouse 5-HT3A subunit have been described previously (Baker et al., 2004). The mammalian cell line tsA201 (derived from the human embryonic kidney 293 cell line) was obtained from Dr. William Green (University of Chicago, Chicago). Cells were cultured in Dulbecco’s modified Eagle’s medium (Invitrogen, Paisley, United Kingdom) containing 2 mM L-GlutaMAX (Invitrogen) plus 10% heat-inactivated fetal calf serum (Sigma, Poole, United Kingdom) with penicillin (100 U/ml) and streptomycin (100 *µ*g/ml) and were maintained in a humidified incubator containing 5% CO_2_ at 37°C. Cells were transiently transfected using Effectene transfection reagent (QIAGEN, Crawley, United Kingdom) according to the manufacturer’s instructions. In all cases, cells were transfected overnight and assayed for expression approximately 40–48 hours after transfection. To ensure that the levels of radioligand binding were not influenced by differences in the amount of subunit cDNA expressed, the amount of each subunit plasmid DNA and also the total amount of plasmid DNA were kept constant when subunits were expressed singly and in combination. This was achieved by the inclusion of empty plasmid expression vector when single subunits were transfected.

Binding studies with [^3^H]-*α*-bungarotoxin (*α*-BTX) in cell membrane preparations were performed essentially as described previously (Lansdell and Millar, 2000; Harkness and Millar, 2002). Membranes (typically, 10–100 *µ*g of protein) were incubated with radioligand (final concentration 20 nM) for 150 minutes at 4°C in a total volume of 300 *µ*l in the presence of protease inhibitors leupeptin (2 *µ*g/ml) and pepstatin (1 *µ*g/ml). Our standard protocol for determining nonspecific binding was the addition of 1 mM carbachol, 1 mM nicotine, and 10 *µ*M methyllycaconitine to triplicate samples. Additional experiments were also performed in which nonspecific binding of [^3^H]-*α*-BTX was determined by displacement of the radioligand by ACh (1 mM). In all cases the levels of specific binding were determined by subtracting the level of nonspecific binding from the total binding (both of which were determined in triplicate). The data were determined as means of three independent experiments, each performed in triplicate. Radioligand binding was assayed by filtration onto 0.5% polyethylenimine-presoaked Whatman GF/B filters (Sigma-Aldrich, Dorset, England) followed by rapid washing (typically, five washes, each of 4 ml) with ice-cold 10 mM phosphate buffer using a Brandel cell harvester, and radioactivity was determined by scintillation counting. Care was taken to ensure that the number of receptor binding sites used for binding studies was low enough to avoid significant (>10%) ligand depletion at low concentrations of radioligand. Preliminary experiments were conducted to ensure that incubation times were long enough to enable radioligand binding to reach equilibrium. Protein concentrations were determined using bovine serum albumin standards (Bio-Rad, Hercules, CA).

#### Molecular Modeling and Docking.

Homology models of the extracellular domain of the chick and rat *α*9*α*10 nAChRs were created with SWISS MODEL (Schwede et al., 2003; Arnold et al., 2006; Bordoli et al., 2009) using the monomeric structure of the human *α*9 subunit as the template (Protein Data Bank ID 4UY2) (http://www.rcsb.org/pdb/explore/explore.do?structureId=4uy2) (Zouridakis et al., 2014). The monomeric models of these proteins were then structurally aligned to the pentameric structure of *Lymnaea stagnalis* AChBP bound to ACh (Protein Data Bank ID 3WIP) (http://www.rcsb.org/pdb/explore/explore.do?structureId=3wip) (Olsen et al., 2014) using the program STAMP (Russell and Barton, 1992) from visual molecular dynamics (Humphrey et al., 1996) to obtain pentameric models with a (*α*9)_2_(*α*10)_3_ stoichiometry bound to ACh. Four different types of possible binding site interfaces were included: *α*9*α*9, *α*9*α*10, *α*10*α*9, and *α*10*α*10. In each interface, the first subunit forms the principal face and the second forms the complementary face. The models were energy minimized to relax steric clashes using spdbviewer (Guex and Peitsch, 1997), and were used for docking studies after deletion of ACh from the models. Using AutoDock version 4.3 (Morris et al., 2009), ACh was docked into each of the four types of interfaces for rat and chick subunits. Two hundred genetic algorithm runs were performed for each condition. Residues R57, R111, and R117 were set as flexible to avoid steric and/or electrostatic effects that may impair ACh docking into the binding site.

Clustering of the results was done with AutoDock based on a root-mean-square deviation cutoff of 2.0 Å. Docking results were corroborated in three different procedures. The most representative docking result was plotted with Discovery Studio Visualizer 3.5 (Accelrys Software, San Diego, CA).

#### Double-Mutant Cycle Analysis.

The EC_50_ values were used to determine the coupling coefficient Ω based on the following equation:
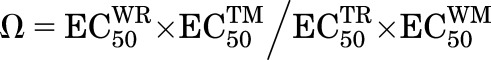
where WR corresponds to wild type; TM corresponds to the double mutant W55TR117M; WM corresponds to the single mutant R117M; and TR corresponds to the single mutant W55T. The coupling energy between residues was calculated by the following equation (Schreiber and Fersht, 1995):


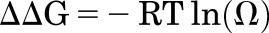


#### Statistical Analysis.

Statistical significance was determined using analysis of variance (ANOVA) followed by the Bonferroni test. Some of our data sets did not fit to a standard Gaussian distribution when tested using Kolmogorov-Smirnov, D’Agostino-Pearson, or Shapiro-Wilk tests. In those cases, statistical significance was evaluated using nonparametric Mann-Whitney or Kruskal-Wallis tests followed by Dunn’s tests. A *P* < 0.05 was considered significant.

All drugs were obtained from Sigma-Aldrich (St. Louis, MO), except when otherwise indicated. ACh chloride was dissolved in distilled water as 100 mM stocks and stored aliquoted at −20°C. 1,2-Bis(2-aminophenoxy)ethane-*N*,*N*,*N*′,*N*′-tetraacetic acid (acetoxymethyl ester) was stored at −20°C as aliquots of a 100 mM solution in dimethylsulfoxide, thawed, and diluted 1000-fold into Barth’s solution shortly before incubation of the oocytes. ACh solutions in Ringer’s saline were freshly prepared immediately before application.

Experiments were carried out in accordance with the Guide for the Care and Use of Laboratory Animals as adopted and promulgated by the U.S. National Institutes of Health (https://grants.nih.gov/grants/olaw/Guide-for-the-Care-and-Use-of-Laboratory-Animals.pdf), and were approved by the Institution’s Animal Care and use Committee.

## Results

### 

#### The Principal Components of *α*9 and *α*10 Subunits Contribute Equally to Function of Rat *α*9*α*10 nAChRs.

To determine the contribution of the principal components of the *α*9 or *α*10 subunits to ligand binding and *α*9*α*10 nAChR function, we generated Y190T mutant subunits (*Torpedo marmorata* *α*1 numbering). Amino acid Y190 is a highly conserved key residue in loop C of *α* nAChR subunits (Karlin, 2002). It has been shown to interact with ACh in a crystal structure of a nAChR homolog from *Lymnaea stagnalis* (Olsen et al., 2014) and with *α*-BTX when crystallized with either the *α*1 (Dellisanti et al., 2007) and *α*9 receptor subunits (Zouridakis et al., 2014) or a *α*7/AChBP chimera (Huang et al., 2013). The substitution of Y190 by threonine profoundly reduces binding and gating of the muscle AChR (Chen et al., 1995) and prevents agonist-evoked responses in human *α*7 and *α*7/5-HT3A receptors (Andersen et al., 2013; Rayes et al., 2009). Additionally, as a consequence of loop C movement during ACh binding stabilization (Gao et al., 2006), Y190 has been reported to disrupt a salt bridge associated with the closed state of the receptor (Mukhtasimova et al., 2005).

**Fig. 1.**
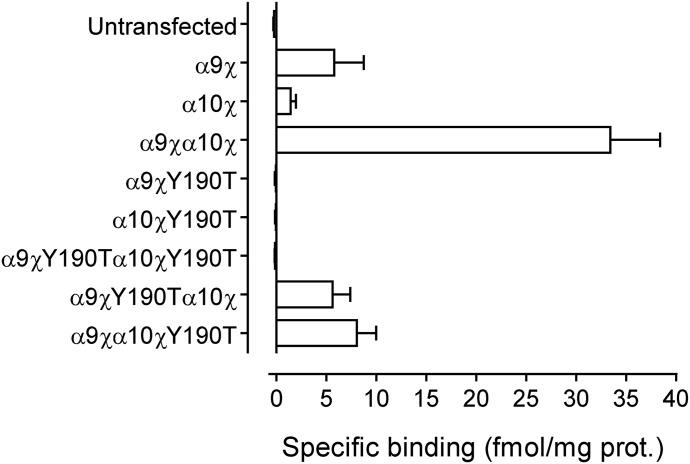
Effect of the Y190T mutation on [^3^H]-*α*-BTX binding. Specific binding levels of [^3^H]-*α*-BTX (final concentration 20 nM) to wild-type and mutated (Y190T) subunit combinations expressed in mammalian tsA201 cells. Data are mean and S.E.M. of three independent experiments, each of which was performed in triplicate.

We first evaluated specific total binding of [^3^H]-*α*-BTX in nAChRs carrying the Y190T mutation. As previously described (Baker et al., 2004), due to undetectable expression levels in cell lines when expressing wild-type *α*9 or *α*10 subunits (Baker et al., 2004), binding studies were performed with chimeric subunits containing the extracellular domain of rat *α*9 or *α*10 subunits fused to the C-terminal domain of the 5-HT3A subunit (referred to as *α*9*χ* and *α*10*χ*, respectively). Specific binding of [^3^H]-*α*-BTX was observed in cells transiently transfected with either *α*9*χ* or *α*10*χ*, indicating membrane targeting of homomeric receptors (Fig. 1). The coexpression of *α*9*χ* and *α*10*χ* resulted in significantly higher levels of [^3^H]-*α*-BTX specific binding, which is likely to be a consequence of more efficient assembly of the chimeric subunits into heteromeric complexes as previously described (Baker et al., 2004). Specific binding of [^3^H]-*α*-BTX to *α*9*χα*10*χ* was 6-fold higher than observed with *α*9*χ* expressed alone (*n* = 3, *P* < 0.0001, Kruskal-Wallis test followed by Dunn’s test).

The introduction of the Y190T substitution into either *α*9*χ* or *α*10*χ* (*α*9*χ*Y190T or *α*10*χ*Y190T) resulted in a complete loss of specific binding of [^3^H]-*α*-BTX when expressed as either homomeric or heteromeric (double-mutant) receptors (Fig. 1). However, when either *α*9*χ*Y190T or *α*10*χ*Y190T was coexpressed with their nonmutated counterpart subunit (*α*9*χ* or *α*10*χ*), specific [^3^H]-*α*-BTX binding was observed, indicating that both *α*9 and *α*10 subunits can contribute to the principal component of the extracellular ligand binding site. Specific binding was 6-fold (*n* = 3) and 4-fold (*n* = 3) lower for *α*9*χ*Y190T*α*10*χ* and *α*9*χα*10*χ*Y190T, respectively, compared with wild-type *α*9*χα*10*χ* (*P* < 0.0001, Kruskal-Wallis test followed by Dunn’s test). However, specific binding of *α*9*χ*Y190T*α*10*χ* was 4-fold higher than that observed for homomeric *α*10*χ* receptors, suggesting that mutant (Y190T) subunits efficiently assemble into heteromeric receptors (*P* = 0.0472, Mann-Whitney test).

**Fig. 2.**
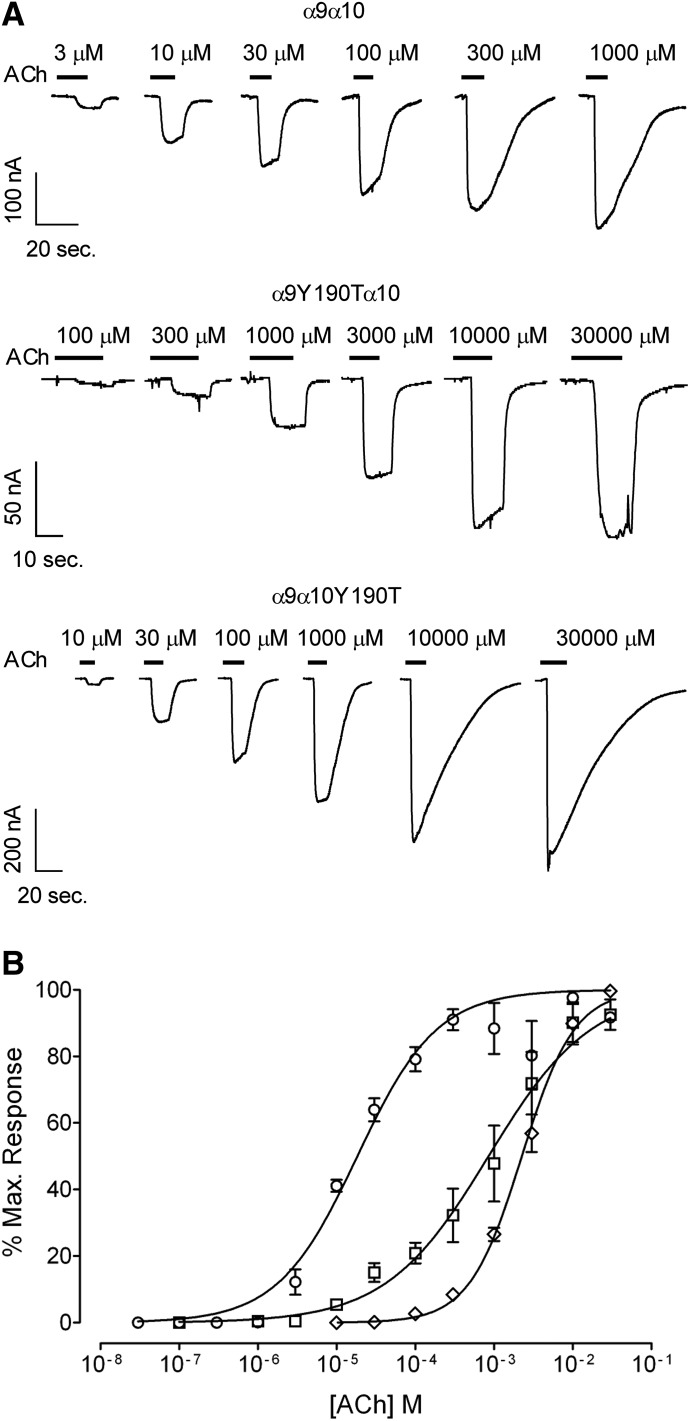
Effect of the Y190T mutation on the response to ACh of rat *α*9*α*10 receptors. (A) Representative traces of responses evoked by increasing ACh concentrations in oocytes expressing wild-type (upper panel), *α*9Y190T*α*10 (middle panel), and *α*9*α*10Y190T (lower panel) receptors. (B) Concentration-response curves to ACh performed in oocytes expressing wild-type (○), *α*9Y190T*α*10 (□), and *α*9*α*10Y190T (◊) receptors. Peak current values were normalized and refer to the maximal peak response to ACh in each case. The mean and S.E.M. of 5–8 experiments per group are shown.

To examine whether Y190T mutants are capable of forming functional channels, receptors were heterogously expressed in *Xenopus laevis* oocytes. Figure 2A shows representative responses to increasing concentrations of ACh for wild-type and Y190T mutant receptors. Both *α*9Y190T*α*10 and *α*9*α*10Y190T complexes formed functional channels. Maximal ACh-evoked currents were similar for wild-type *α*9*α*10 and *α*9*α*10Y190T mutants (Table 1) and were an order of magnitude larger than those previously reported for *α*9 homomeric receptors (Elgoyhen et al., 2001), indicating that the resultant responses are not due to the expression of *α*9 homomeric wild-type receptors. Moreover, responses of *α*9Y190T*α*10 receptors derive from the incorporation of *α*9Y190T mutant subunits to the heteromeric receptor since *α*9Y190T homomeric receptors lack functional ligand binding sites (Fig. 1) and rat and human *α*10 homomers are nonfunctional (Elgoyhen et al., 2001; Sgard et al., 2002). Double-mutant *α*9Y190T*α*10Y190T receptors failed to respond to either 1 or 30 mM ACh (*n* = 8), a result consistent with the lack of binding sites (Fig. 1). As displayed in Fig. 2B, the Y190T substitution in either *α*9 or *α*10 produced a shift of the ACh concentration-response curve to the right and an increase in the ACh EC_50_ of two orders of magnitude (Table 1). The increase in the EC_50_ of *α*9*α*10Y190T mutant receptors compared with wild-type receptors once again indicates that Y190T mutants are assembly competent, and that responses do not derive from homomeric *α*9 wild-type receptors. Taken together, these results suggest that both *α*9 and *α*10 can contribute with their principal components to the binding site and that the integrity of both is necessary for wild-type receptor function.

**TABLE 1:** Maximal evoked currents and concentration-response curve parameters The number of experiments (*n*) represents independent oocytes from 3 to 6 different frogs. Asterisks (*) indicate the results are significantly different from the control wild-type *α*9*α*10. Comparisons of EC_50_ values for wild-type, mutant *α*9, mutant *α*10, or double-mutant receptors for each mutated residue were performed with one-way ANOVA followed by the Bonferroni test.

Species	Receptor	*I*_max_	EC_50_	*p*	*n*
		*nA*	*µM*		
Rat	*α*9*α*10	298 ± 48	18 ± 3		8
	*α*9Y190T*α*10	112 ± 6	2254 ± 155	<0.0001*	5
	*α*9*α*10Y190T	336 ± 91	850 ± 170	<0.0001*	6
	*α*9CC/SS*α*10	402 ± 103	148 ± 9	<0.0001*	8
	*α*9*α*10CC/SS	571 ± 113	147 ± 17	<0.0001*	17
	*α*9CC/SS*α*10CC/SS	360 ± 119	405 ± 13	<0.0001*	6
	*α*9W55T*α*10	42 ± 4	1022 ± 35	<0.0001*	5
	*α*9*α*10W55T	177 ± 81	36 ± 1	0.0665	6
	*α*9*α*10R117M	107 ± 38	31 ± 5	0.0655	5
	*α*9*α*10 W55T/R117M	245 ± 83	768 ± 135	0.0011*	11
Chicken	*α*9*α*10	100 ± 12	16 ± 2		6
	*α*9W55T*α*10	59 ± 8	357 ± 75	<0.0001*	6
	*α*9*α*10W55T	159 ± 32	334 ± 13	<0.0001*	6

**Fig. 3.**
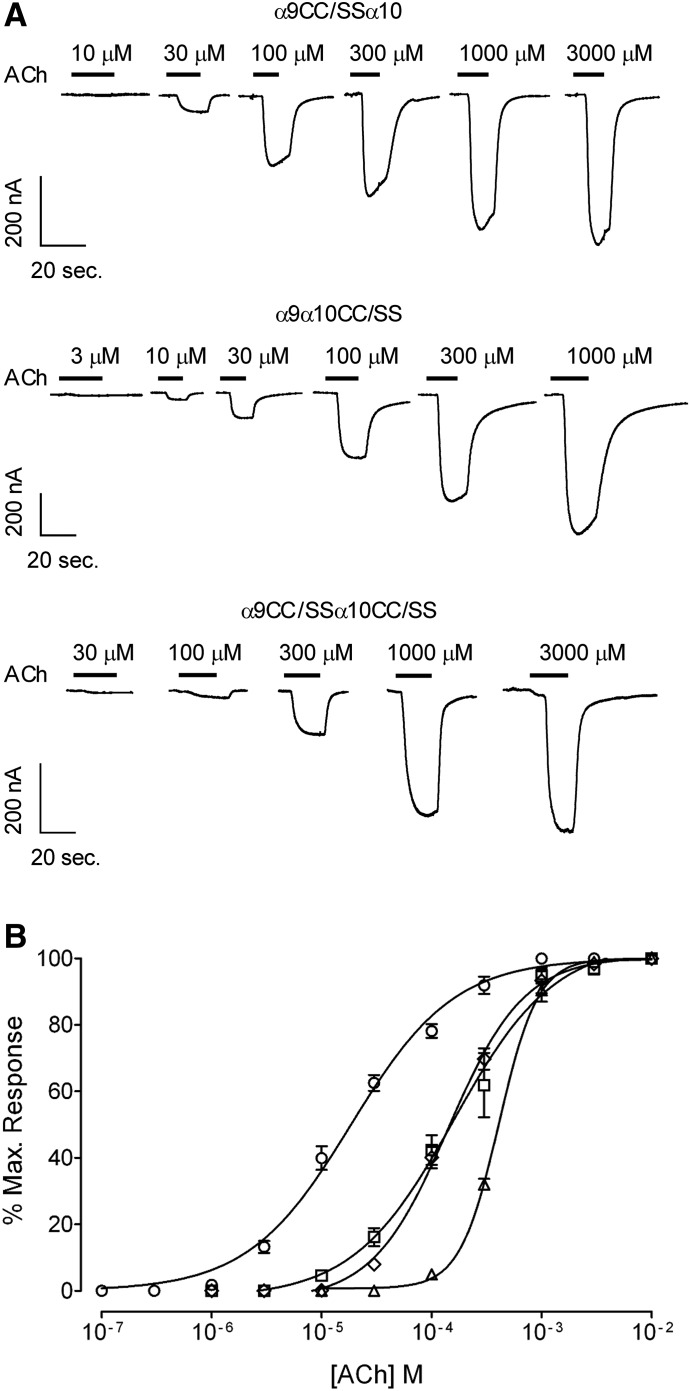
Effect of the CC192/193SS (CC/SS) mutations on the response to ACh of rat *α*9*α*10 receptors. (A) Representative traces of responses evoked by increasing ACh concentrations in oocytes expressing *α*9CC/SS*α*10 (upper panel), *α*9*α*10CC/SS (middle panel), and *α*9CC/SS*α*10CC/SS (lower panel) receptors. (B) Concentration-response curves to ACh performed in oocytes expressing wild-type (○), *α*9CC/SS*α*10 (□), *α*9*α*10CC/SS (◊), and *α*9CC/SS*α*10CC/SS (△) receptors. Peak current values were normalized and refer to the maximal peak response to ACh in each case. The mean and S.E.M. of 6–17 experiments per group are shown.

To further analyze the participation of the principal components of both *α*9 and *α*10 to receptor function, we mutated to serine the double cysteines of loop C, C192S/C193S [i.e., double cysteine to serine (CC/SS)], a hallmark of nAChR *α* subunits (Karlin, 2002). Figure 3A shows representative responses to increasing concentrations of ACh evoked in *Xenopus laevis* oocytes expressing mutant receptors bearing the CC/SS substitution in either *α*9 or *α*10 subunits, or both. Surprisingly, the CC/SS double-mutant receptors were functional. The CC/SS substitution in either *α*9 or *α*10 produced a similar shift of the ACh concentration-response curve to the right and an increase in the ACh EC_50_ of one order of magnitude (EC_50_: wild type = 18 ± 3 *µ*M; *α*9CC/SS*α*10 = 148 ± 9 *µ*M, *n* = 8, *P* < 0.0001; *α*9*α*10CC/SS = 147 ± 17 *µ*M, *n* = 17, *P* < 0.0001, one-way ANOVA followed by the Bonferroni test) (Fig. 3B; Table 1). Further shift of the concentration-response curve and an increase of the ACh EC_50_ were observed in double-mutant CC/SS receptors (405 ± 13 *µ*M, *n* = 6, *P* < 0.0001 compared with wild type, one-way ANOVA followed by the Bonferroni test).

#### Nonequivalent Contribution of *α*9 and *α*10 Complementary Components to Rat *α*9*α*10 nAChR Receptor Function.

To determine the contribution of the complementary faces of either *α*9 or *α*10 to rat *α*9*α*10 nAChR function, we generated W55T mutant subunits. Amino acid W55 is highly conserved within loop D of nAChR subunits, which contributes to the complementary face of the ligand binding site (Karlin, 2002). The crystal structure of the ACh binding protein from *Lymnaea stagnalis* bound to ACh shows a cation-*π* interaction of W55 with this agonist (Olsen et al., 2014). Moreover, the substitution of W55 by threonine in an *α*7/5-HT3A chimera renders a receptor that binds *α*-BTX but impairs competition of [^3^H]-*α*-BTX by ACh, leading to nonfunctional receptors (Rayes et al., 2009). In addition, mutagenesis analysis in the *Torpedo* electric organ nAChR has demonstrated that W55 is part of the ACh binding pocket of nAChRs (Xie and Cohen, 2001).

**Fig. 4.**
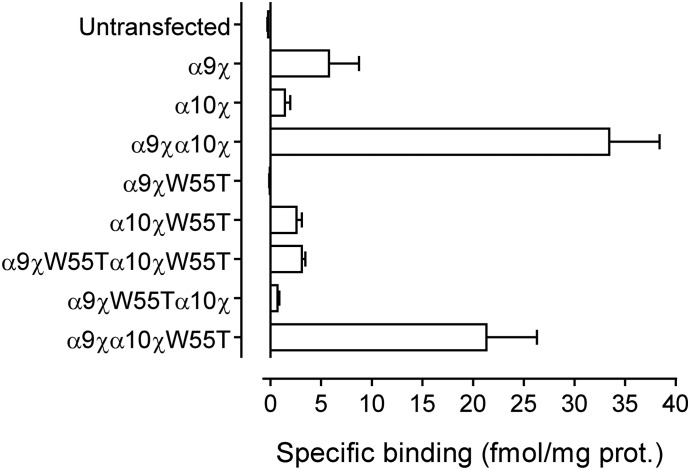
Effect of the W55T mutation on [^3^H]-*α*-BTX binding. Specific binding of [^3^H]-*α*-BTX (final concentration 20 nM) to wild-type and mutated (W55T) subunit combinations expressed in mammalian tsA201 cells. Data are mean and S.E.M. of three independent experiments, each of which was performed in triplicate.

Figure 4 shows binding experiments performed with [^3^H]-*α*-BTX in wild-type and W55T mutant *α*9*α*10 receptors. In contrast to previous findings reported for the *α*7/5-HT3A subunit chimera (Rayes et al., 2009), no detectable specific binding was observed with homomeric *α*9*χ*W55T receptors. In contrast, homomeric *α*10*χ*W55T receptors showed significant levels of specific binding, similar to levels observed with homomeric *α*10*χ* (2.5 ± 0.6 and 1.4 ± 0.5 fmol/mg, respectively, *P* = 0.229, Mann-Whitney test). Consistent with these results, heteromeric receptors containing a mutant *α*9*χ*W55T subunit (*α*9*χ*W55T*α*10*χ* and *α*9*χ*W55T*α*10*χ*W55T) showed binding levels similar to those observed with either *α*10*χ* or *α*10*χ*W55T when expressed alone (*P* = 0.1–0.7, Mann-Whitney test). Moreover, receptors composed of wild-type *α*9*χ* subunits and mutated 10*χ* (*α*9*χα*10*χ*W55T) displayed specific binding levels similar to those observed with wild-type heteromeric *α*9*χα*10*χ* receptors (*P* = 0.114, Mann-Whitney test). Taken together, these results indicate that the conserved amino acid W55 in loop D is involved in the binding site of the *α*9*α*10 receptor only when provided by the *α*9 subunit. This appears to suggest that the *α*9 subunit contributes to the complementary component of the binding site of *α*9*α*10 nAChRs and that the (−) faces of *α*9 and *α*10 are nonequivalent.

An important question is whether ACh binds to *α*9*χα*10*χ*W55T receptors. To discriminate between total and specific binding of [^3^H]-*α*-BTX we used a standard protocol in which a mixture of cold ligands were used to determine nonspecific binding. To confirm whether ACh itself is able to displace binding of [^3^H]-*α*-BTX we repeated these binding experiments and used only ACh to displace bound [^3^H]-*α*-BTX. For both wild-type (*α*9*χα*10*χ*) and mutated (*α*9*χα*10*χ*W55T) nAChRs, bound [^3^H]-*α*-BTX was displaced as efficiently with ACh alone as with our standard mixture of nonradioactive competing ligands, confirming that the ACh binding site is retained in *α*9*χα*10*χ*W55T. This indicates that the W55 mutation has a different effect in *α*10 to that observed with the *α*9 subunit and its previously reported effect in *α*7 (Rayes et al., 2009), and suggests that W55 contributes differently to the ACh binding site of the *α*9*α*10 receptor when provided by the *α*9 or *α*10 subunit. Since W55 is a highly conserved key residue present in loop D of nicotinic subunits that contributes to complementary components of binding sites (Karlin, 2002), the present results are consistent with the conclusion that *α*10 either does not contribute to the (−) face of the binding site of the *α*9*α*10 receptor or that W55 of *α*10 is not readily accessible within the binding pocket. If the latter is the case, then the contributions of the (−) faces of *α*9 and *α*10 to the binding interface are nonequivalent. To further examine these possibilities, the functional responses of W55T mutated receptors were studied in *Xenopus laevis* oocytes.

**Fig. 5.**
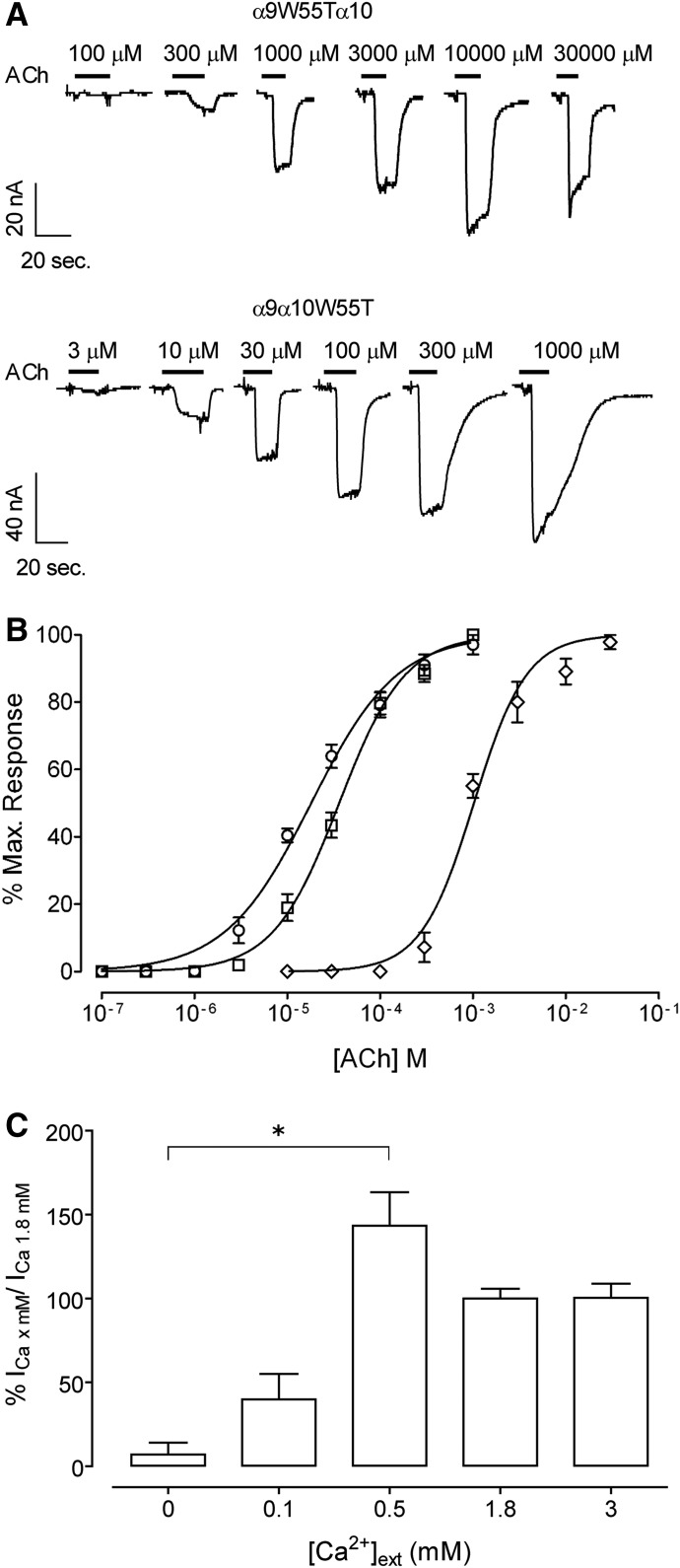
Effect of the W55T mutation on the response to ACh of rat *α*9*α*10 receptors. (A) Representative traces of responses evoked by increasing ACh concentrations in oocytes expressing *α*9W55T*α*10 (upper panel) and *α*9*α*10W55T (lower panel) receptors. (B) Concentration-response curves to ACh performed in oocytes expressing wild-type (○), *α*9W55T*α*10 (□), and *α*9*α*10W55T (◊) receptors. Peak current values were normalized and refer to the maximal peak response to ACh. The mean and S.E.M. of 5–8 experiments per group are shown. (C) Bar diagram illustrating the modulation of the *α*9*α*10W55T receptor by extracellular Ca^2+^ exerts. Current amplitudes obtained at different Ca^2+^ concentrations in each oocyte were normalized with respect to that obtained at 1.8 mM in the same oocyte. The mean and S.E.M. of three experiments per group are shown.

Figure 5A shows representative responses to increasing concentrations of ACh in *Xenopus laevis* oocytes expressing wild-type rat *α*9*α*10 receptors or W55T mutant receptors. Double-mutant *α*9*α*10 receptors failed to evoke currents at 1 or 30 mM ACh (*n* = 15). The W55T substitution in *α*9 produced a displacement of the concentration-response curve to ACh to the right with a 60-fold increase in the EC_50_ (EC_50_: wild type = 18 ± 3 *µ*M, *α*9W55T*α*10 = 1022 ± 35 *µ*M, *P* < 0.0001, one-way ANOVA followed by the Bonferroni test, *n* = 5–8) (Table 1). On the other hand, the W55T substitution in *α*10 produced only a slight (although nonsignificant) increase in the receptor EC_50_ (EC_50_: wild type = 18 ± 3 *µ*M, *α*9*α*10W55T = 36 ± 1 *µ*M, *P* = 0.0665 one-way ANOVA followed by the Bonferroni test, *n* = 6) (Table 1). Maximal evoked currents of *α*9*α*10W55T receptors were not significantly different from those of wild-type *α*9*α*10 receptors (*I*_max_: wild type = 298 ± 48 nA, *α*9*α*10W55T = 177 ± 81 nA, *P* = 0.1826, Mann-Whitney test, *n* = 6) (Table 1) and one order of magnitude larger than those reported for *α*9 homomeric receptors (Rothlin et al., 1999; Katz et al., 2000), indicating that *α*10W55T is incorporated into a *α*9*α*10W55T heteromeric receptor.

To further rule out the possibility that the modest effect observed in responses to ACh of *α*9*α*10W55T receptors is due to the lack of incorporation of the *α*10W55T subunit into a heteromeric assembly, we analyzed the Ca^2+^ sensitivity of the resultant receptors. Homomeric *α*9 receptors are only blocked by extracellular Ca^2+^, whereas heteromeric *α*9*α*10 receptors are potentiated in the submillimolar range and blocked at higher concentrations of this divalent cation (Katz et al., 2000; Weisstaub et al., 2002). Figure 5C shows the modulation profile obtained at a concentration of ACh close to the EC_50_ (30 *µ*M) value and the application of increasing concentrations of extracellular Ca^2+^. Peak current amplitudes at each Ca^2+^ concentration in each oocyte were normalized to those obtained at 1.8 mM. Similar to that reported for wild-type receptors (Elgoyhen et al., 2001; Weisstaub et al., 2002), a biphasic Ca^2+^ modulation profile was observed with maximal responses at 0.5 mM. A one-way ANOVA followed by multiple comparisons indicated that the difference in normalized mean current amplitude between nominal 0 and 0.5 mM Ca^2+^ is significant (*P* = 0.019, Kruskal-Wallis test followed by Dunn’s test). This result demonstrates the occurrence of Ca^2+^ potentiation and thus confirms the incorporation of *α*10W55T subunits into pentameric receptors.

The functional results indicate that both *α*9 and *α*10 contribute to the (−) face of the intersubunit interface, but that their contribution is nonequivalent. Thus, if *α*10 did not contribute at all to the (−) face, the shift in the ACh concentration-response curve of double-mutated W55T receptors should resemble that of *α*9W55T receptors instead of rendering nonfunctional receptors (Fig. 5B).

#### The *α*9 and *α*10 Subunits Contribute Equally to the Complementary Component of the ACh Binding Site in the Chicken *α*9*α*10 nAChR.

**Fig. 6.**
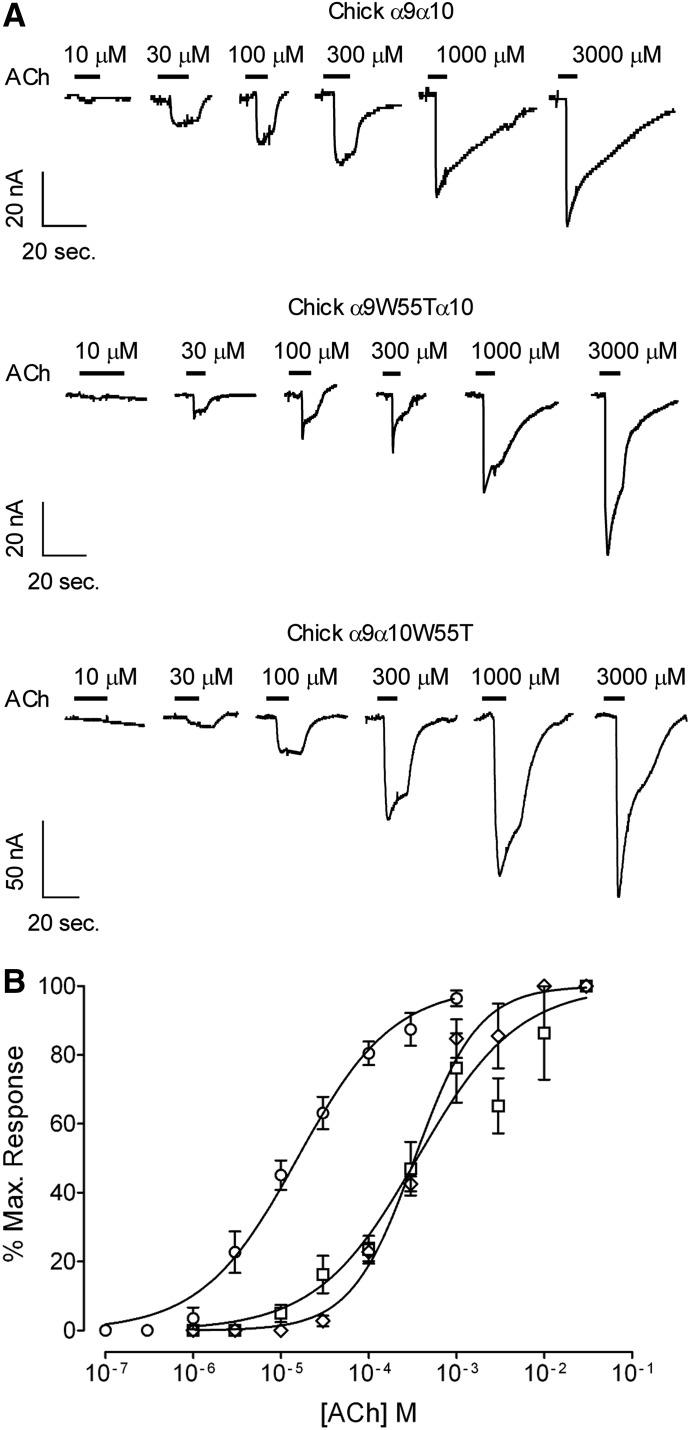
Effect of the W55T mutation on the response to ACh of chicken *α*9*α*10 receptors. (A) Representative traces of responses evoked by increasing ACh concentrations in oocytes expressing wild-type (upper panel), *α*9W55T*α*10 (middle panel), and *α*9*α*10W55T (lower panel) chick receptors. (B) Concentration-response curves to ACh performed in oocytes expressing wild-type (○), *α*9W55T*α*10 (□), and *α*9*α*10W55T (◊) chick receptors. Peak current values were normalized and refer to the maximal peak response to ACh. The mean and S.E.M. of six experiments per group are shown.

The asymmetric contribution of *α*9 and *α*10 subunits to the (−) face of the ACh binding site might result from the adaptive evolution that occurred only in mammalian *CHRNA10* genes. This resulted in important nonsynonymous amino acid substitutions in the coding region of the *α*10 nAChR subunits, including that of loop D (Franchini and Elgoyhen, 2006; Elgoyhen and Franchini, 2011; Lipovsek et al., 2012). If this were the case, then both *α*9 and *α*10 should equally contribute to the (−) face of the intersubunit interface in a nonmammalian vertebrate species. Figure 6A shows representative responses to increasing concentrations of ACh evoked in *Xenopus laevis* oocytes expressing chicken *α*9*α*10 wild-type and W55T mutant receptors. Double-mutant receptors failed to evoke currents at 1 or 30 mM ACh (*n* = 10). The W55T substitution in either *α*9 or *α*10 produced similar shifts in the ACh concentration-response curves to the right (Fig. 6) and a one order of magnitude increase in the receptor EC_50_ (EC_50_: wild type = 16 ± 2 *µ*M, *α*9W55T*α*10 = 357 ± 75 *µ*M, *α*9*α*10W55T = 334 ± 13 *µ*M, *P* < 0.0001, one-way ANOVA followed by the Bonferroni test, *n* = 6) (Table 1). This result suggests that, in contrast to the situation with rat *α*9*α*10 receptors, in chicken the (−) face of both *α*9 and *α*10 subunits equally contribute to receptor function.

#### Molecular Docking of ACh in *α*9*α*10 Receptors.

**Fig. 7.**
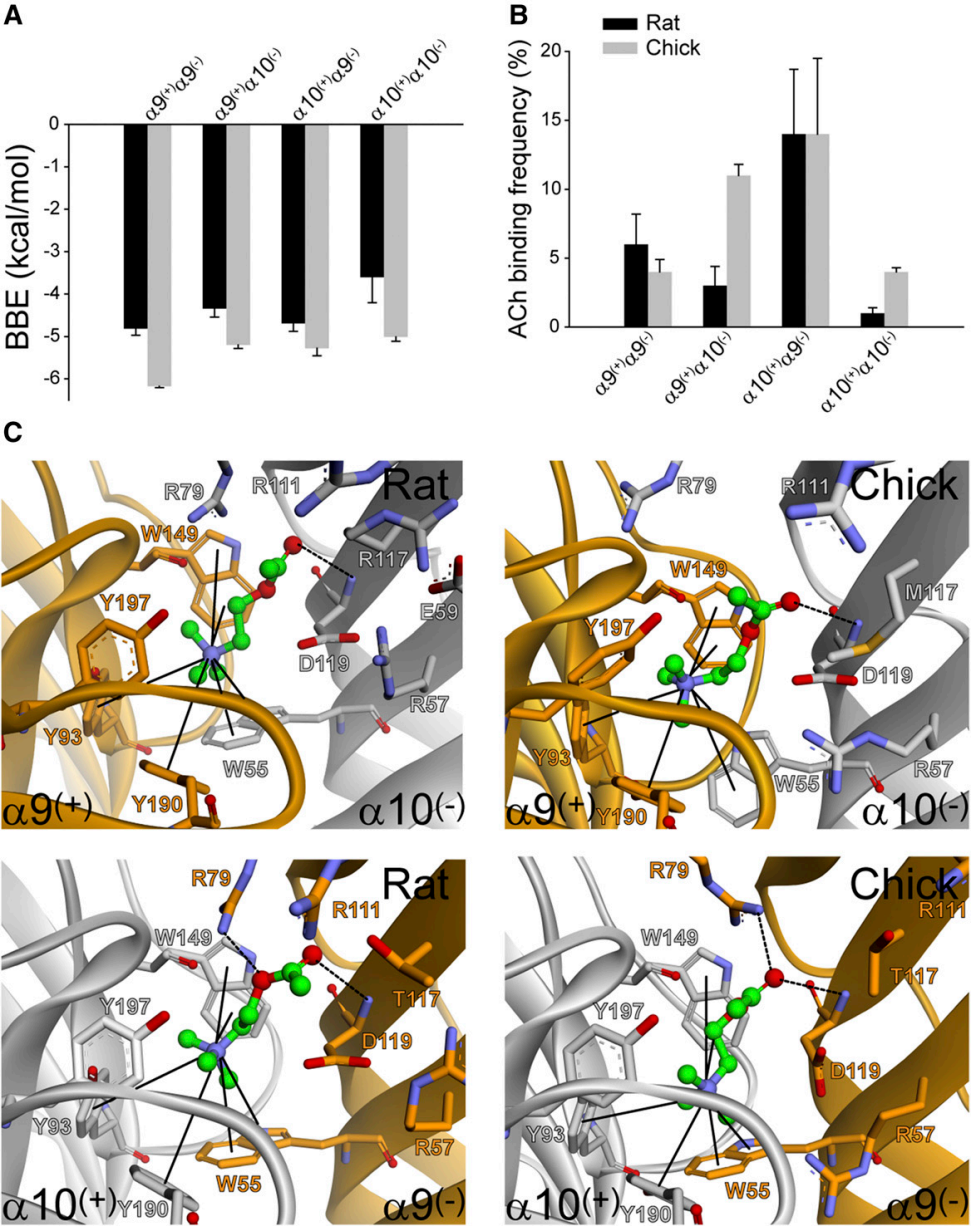
Docking of ACh into homology-modeled *α*9/*α*10 binding-site interfaces. ACh was docked in the correct orientation into the two possible models for heteromeric interfaces of rat and chicken receptors. The BBE (A) and the percentage of favorable conformations (B) for bound ACh were averaged from three different runs for each interface. (C) Representative models of ACh docked into the different interfaces. The main *π*-cation interactions are shown with straight lines and the H-bonds are shown with dashed lines.

To gain further insight into the contribution of the subunit components to ACh binding, we modeled different subunit arrangements to take into account the four possible subunit interfaces [*α*9(+)*α*9(−), *α*9(+)*α*10(−), *α*10(+)*α*10(−), and *α*10(+)*α*9(−)] in rat and chicken receptors, and performed molecular docking studies. To evaluate the capability of each interface to bind ACh, we compared the best binding energy (BBE) (Fig. 7A) and the frequency of conformations that bind the agonist in the correct orientation in the binding pocket (Fig. 7B). For all interfaces, the conformations considered as favorable were those showing the previously described cation-*π* interactions between the amino group of ACh and aromatic residues of the binding pocket (W55, Y93, W149, and Y190) (Dougherty, 2007; Hernando et al., 2012) (Fig. 7C). In these conformations, and for all interfaces, ACh shows the capability to form hydrogen bonds with D119 and Y197, which are equivalent to conserved H bonds of different nAChRs (Tomaselli et al., 1991; Lester et al., 2004; Hernando et al., 2012) (Fig. 7C). The BBE did not show important differences among the different models, except for the homomeric rat *α*10*α*10 interface. At this interface, the BBE was about −3.5 kcal/mol compared with −5 to −6 kcal/mol for all of the others (Fig. 7A).

The main difference in the docking results among interfaces was detected in the frequency of favorable conformations (Fig. 7B). In rat, the most frequent conformations with ACh in the correct orientation at the binding site was observed at the interface in which *α*10 contributes to the principal and *α*9 to the complementary face [*α*10(+)*α*9(−) interface], with a BBE of −4.8 kcal/mol (Fig. 7). Models with rat the *α*10 subunit placed in the complementary face [*α*9(+)*α*10(−) or *α*10(+)*α*10(−)] showed a significant reduction of the frequency of conformations with ACh docked in the correct orientation (Fig. 7B). In the case of *α*10(+)*α*10(−), ACh only showed a favorable orientation at the binding site in less than 2% of the conformations in most of the docking conformations (Fig. 7B).

In chicken heteromeric interfaces, no significant differences were observed in the frequency of favorable conformations between the *α*9(+)*α*10(−) and *α*10(+)*α*9(−) interfaces. Thus, in contrast to the rat nAChR, this suggests that *α*10 contributes similarly to both the principal and complementary faces of the chicken receptor (Fig. 7). When comparing homomeric interfaces, rat *α*10(+)*α*10(−) appears to be very unfavorable for ACh binding (i.e., the lowest frequency of conformations with ACh in the correct orientation and the highest BBE). In chicken, both homomeric interfaces appear to be similarly favorable for ACh binding, but less favorable than the heteromeric ones (Fig. 7).

Taken together, the in silico studies support the experimental data indicating that in rat the contribution of *α*9 and *α*10 to complementary components is nonequivalent. In contrast, *α*9 can form relatively appropriate interfaces for ACh binding when placed at either the principal or complementary faces. Moreover, the modeling supports the functional data for chicken receptors, where *α*10 equally contributes to principal and complementary components.

#### *α*10 Residue 117 in Loop E of the (−) Face Is a Major Determinant of Functional Differences.

**Fig. 8.**
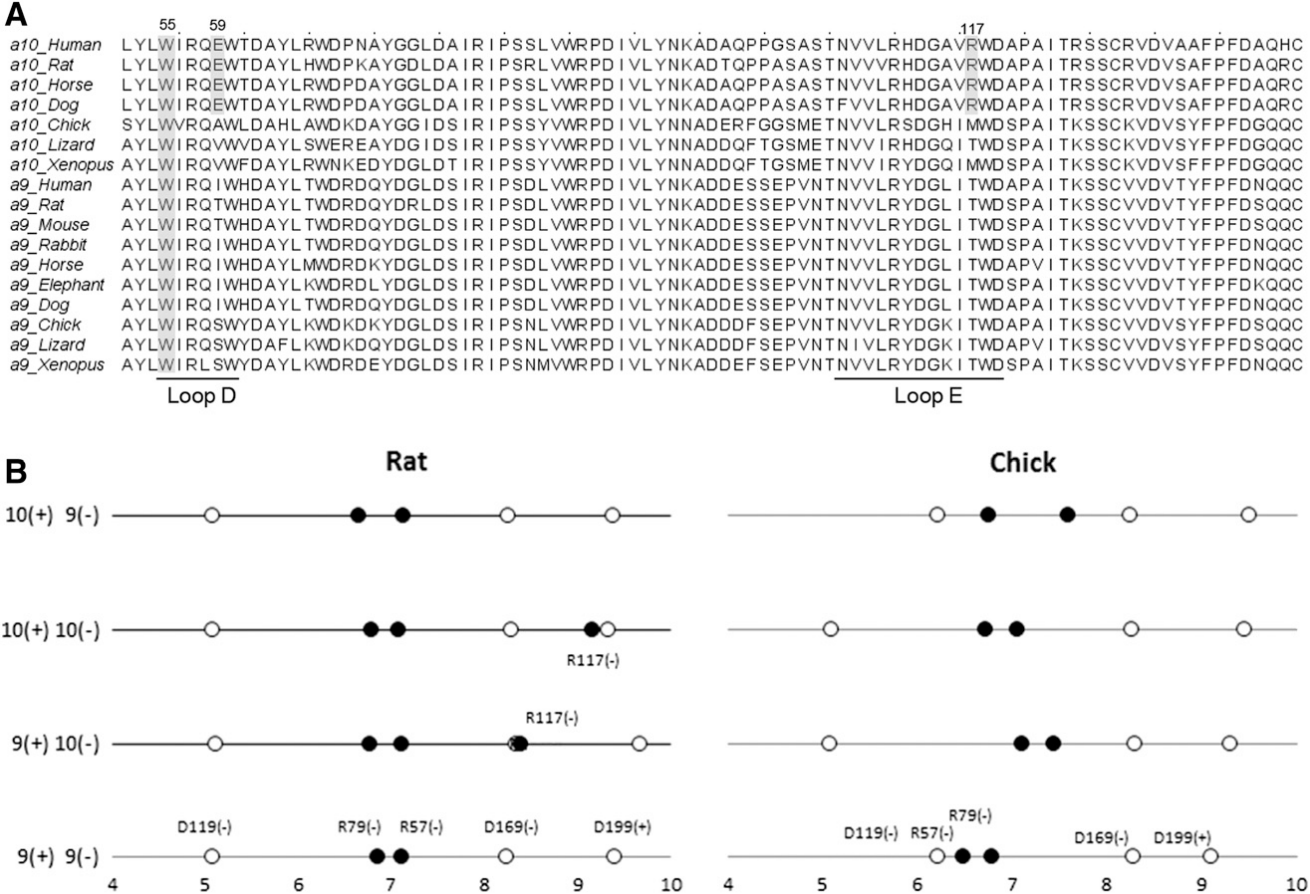
The *α*9 and *α*10 subunit sequence alignments and distribution of charged residues. (A) Sequence alignments of part of the (−) face of *α*9 and *α*10 from different vertebrate species. Conserved W55 and mammalian positively selected E59 and R117 are shaded. (B) Distance (Å) of protein charged groups from the nitrogen atom of ACh in chicken and rat receptors. The analysis was made using the theoretical models constructed by homology modeling described in *Materials and Methods*. The results are shown for the four types of interfaces: *α*9(+)*α*9(−), *α*9(+)*α*10(−), *α*10(+)*α*10(−), and *α*10(+)*α*9(−). Positively charged groups are represented by black circles, whereas the negatively charged groups are represented by white circles. The identity of each residue is shown.

Given that the main key interactions at the binding site with aromatic residues are conserved in all models in conformations where ACh is bound in the correct orientation (Fig. 7), we analyzed in more detail other residues that might account for the fact that W55 is not a major determinant of rat *α*10 subunit complementary components, compared with rat *α*9 and chicken *α*9 and *α*10. Analysis of the model of ACh bound to the four different types of interfaces [*α*9(+)*α*9(−), *α*9(+)*α*10(−), *α*10(+)*α*10(−), and *α*10(+)*α*9(−)] shows that the residues on a radial distribution of 5 Å are the same for the principal components (Y93, S148, W149, Y190, C192, and Y197) and for most of the complementary components (W55, R57, R79, N107, V109, T/M/R117, and D119). They only differ at position 117, where the rat *α*10 positively charged arginine (R117) which is highly conserved in mammalian *α*10 subunits, is substituted by a noncharged methionine in chicken *α*10 and a threonine or methionine in nonmammalian *α*10 subunits (Figs. 7A and 8A); for an extended number of species see Lipovsek et al. (2012, 2014). Interestingly, all *α*9 subunits carry a threonine at this position. Moreover, the appearance of the R117 nonsynonymous amino acid substitution in mammalian species has been under positive selection pressure (Franchini and Elgoyhen, 2006). In many docking conformations R117 was placed toward the cavity (Fig. 7C). Moreover, R117 had to be set as flexible to avoid steric and/or electrostatic effects that impair ACh docking into the correct binding site (see *Materials and Methods*). In addition, rat *α*10 subunits have a negatively charged glutamic acid residue E59 in loop D, which is highly conserved and has been also positively selected in mammalian species (Franchini and Elgoyhen, 2006), compared with noncharged residues in nonmammalian *α*10 and *α*9 subunits (Fig. 8A).

Because R117 and E59 are charged residues, due to the long-range nature of electrostatic interactions, we analyzed the distance distribution of protein-charged groups from the positively charged N atom of ACh (Fig. 8B). In all interfaces, the conserved residues observed on a radial distribution of 10 Å from this N atom were D119(−), R57(−), R79(−), D169(−), and D199(+) in order of increasing distance. Here, the plus and minus signs correspond to the presence of residues in either the principal (+) or complementary (−) face, respectively, and not to the charge of each residue. The most significant difference was the positively charged R117 at a distance of ∼8 to 9 Å from the ACh amino group, which was only present in the complementary site of rat *α*10. This relative excess in positively charged residues in rat *α*10 could result in an unfavorable interaction with the ligand through electrostatic repulsion and thus may perturb the binding site. Interestingly, the negatively charged E59 is close to R117. Although this residue could partially compensate for the positive charge of R117, it is located more than 10 Å from ACh, and thus its effect on the ligand is lower than that of R117. Moreover, the analysis of positively and negatively charged residues in the entire N-terminal domain of rat and chick subunits indicates that the global balance is neutral in rat *α*10, whereas it is strongly negative in rat *α*9 and chicken *α*9 and *α*10 subunits. The difference is due to an excess of basic residues (R and K) in rat *α*10 compared with the other subunits (Table 2). Overall, these observations further confirm that the complementary faces of rat *α*9 and *α*10 subunits are nonequivalent and that R117 in the complementary component of *α*10 might account for functional differences.

**TABLE 2:** Number of charged residues in rat and chicken *α*9 and *α*10 subunits The basic-acidic balance was calculated as the difference in the number of basic (R and K) compared with acidic (D and E) amino acid residues.

Species	Subunit	Acidic (D and E)	Basic (R and K)	Basic-Acidic Balance
Rat	*α*9	34	16	−18
	*α*10	24	24	0
Chick	*α*9	33	18	−15
	*α*10	28	18	−10

**Fig. 9.**
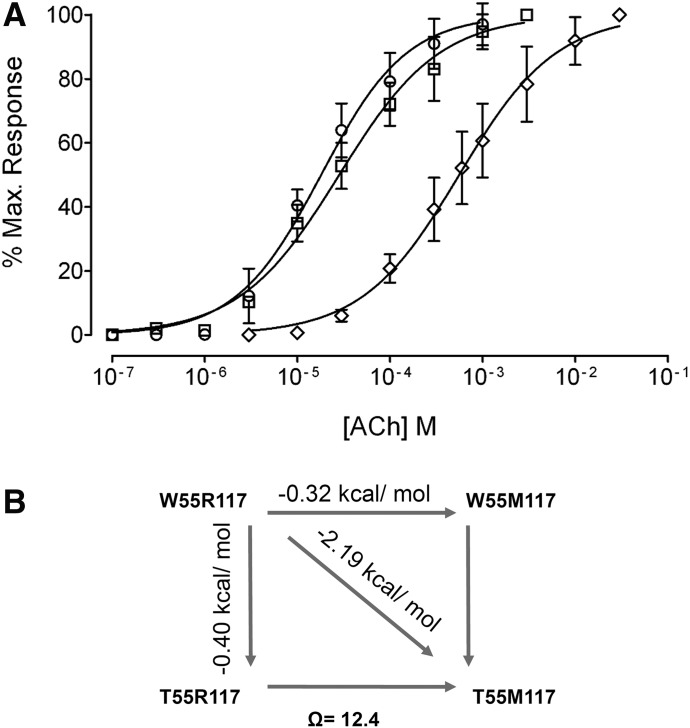
Effect of the R117M mutation on rat *α*9*α*10 receptors. (A) Concentration-response curves to ACh performed in oocytes expressing wild-type (○), *α*9*α*10R117M (□), and *α*9*α*10W55T/R117M (◊) double-mutant rat receptors. Peak current values were normalized and refer to the maximal peak response to ACh. The mean and S.E.M. of 5–11 experiments per group are shown. (B) Scheme for double-mutant cycle analysis. ΔΔG values corresponding to each mutant are shown. These values were calculated as −RTln(EC_50_ mutant/EC_50_ wild type). The coupling parameter, Ω, was calculated as indicated in *Materials and Methods*.

We introduced the R117M substitution in the rat *α*10 subunit and expressed it in *Xenopus* oocytes with rat *α*9 (Fig. 9A). The *α*9*α*10R117M receptors were functional and their ACh EC_50_ values, although slightly higher, did not significantly differ from that of wild-type receptors (Table 1). However, when W55 of *α*10R117M subunits was mutated to threonine, a 43-fold shift in the ACh concentration-response curve to the right was observed (EC_50_: wild type = 18 ± 3 *µ*M, *α*9*α*10 W55T/R117M = 768 ± 135 *µ*M, *P* = 0.0011, one-way ANOVA followed by the Bonferroni test, *n* = 5–11) (Fig. 9; Table 1). Thus, it appears that when the R117 is removed, W55 contributes to the (−) face of rat *α*10 subunits.

The typical way to analyze a system in which two mutations are evaluated individually and in tandem is by mutant cycle analysis (Schreiber and Fersht, 1995; Corradi et al., 2007). Such analysis reveals whether the contributions from a pair of residues are additive or if the effects of mutations are coupled. We calculated the changes due to R117M and W55T mutations in the free energy of the responses using the EC_50_ values (Fig. 9B). Single-mutants *α*10W55T and *α*10R117M decreased the free energy (−0.40 and −0.32 kcal/mol, respectively); the change in the free energy of the double mutant was significantly different from the sum of the changes occurring in the two single mutants (−2.19 kcal/mol). To quantify energetic coupling between *α*10W55 and *α*10R117 we analyzed the changes in the free energy of coupling by double-mutant thermodynamic cycles. When the EC_50_ values are cast as a mutant cycle, the coupling coefficient is 12.4, which corresponds to free energy coupling of −1.47 kcal/mol. Taken together these results indicate that the effects of the mutations are not independent and that the residues are coupled in their contribution to function (Schreiber and Fersht, 1995; Corradi et al., 2007).

## Discussion

The present study shows that, contrary to previous assumptions, the *α*10 subunit contributes to the principal face of the ligand binding site in the heteromeric *α*9*α*10 nAChR. Moreover, we show that the contribution of rat *α*9 and *α*10 subunits to the complementary face is nonequivalent. It is worth noting that conotoxin RgIA, which potently blocks *α*9*α*10 nAChRs (Ellison et al., 2006), was initially reported to bind to the *α*9(+)*α*10(−) interface based on molecular modeling, docking, and molecular dynamics simulations (Pérez et al., 2009). However, mutagenesis experiments have shown that conotoxins RgIA (Azam and McIntosh, 2012; Azam et al., 2015) and Vc1.1 (Yu et al., 2013) bind to the *α*10(+)*α*9(−) interface, further indicating that *α*10 contributes to the principal component of the binding site for antagonist as well as agonist binding.

The lack of [^3^H]-*α*-BTX binding to homomeric (*α*9*χ*Y190T and *α*10*χ*Y190T) and heteromeric (*α*9*χ*Y190T*α*10*χ*Y190T) nAChRs is in agreement with the observation that Y190 in loop C of the principal component interacts with *α*-BTX when crystallized with either the *α*1 (Dellisanti et al., 2007), *α*9 (Zouridakis et al., 2014), or an *α*7/AChBP chimera (Huang et al., 2013). Moreover, Y190 has been shown to interact with ACh in a crystal structure of a nAChR homolog from *Lymnaea stagnalis* (Olsen et al., 2014). Therefore, the lack of binding of [^3^H]-*α*-BTX to Y190T mutant receptors most likely also indicates disrupted ACh binding sites. These binding experiments with Y190T mutated receptors, together with the expression studies, indicate that both *α*9 and *α*10 can contribute to the principal component of the agonist binding site.

The fact that the mutation of the CC/SS mutant, a hallmark of nAChR *α* subunits, in either *α*9 or *α*10 produced similar rightward shifts in the concentration-response curves to ACh further indicates that both subunits can equally contribute to the principal components of the binding site. The observation that *α*9CC/SS*α*10CC/SS double-mutant receptors were functional, albeit with a further increase in the ACh EC_50_ value, indicates that the ACh binding pocket is not completely disrupted in the absence of the continuous double cysteines of the principal component. This is in line with the observation that in the crystal structure of the *Lymnaea stagnalis* nAChR bound to ACh this agonist is wedged in between the disulfide bridge of the double cysteine, but that interactions occur with aromatic residues (Olsen et al., 2014). Likewise, mutation of the CC in the *Aplysia californica* AChBP produces a 10-fold decrease in affinity but does not abolish ACh binding (Hansen and Taylor, 2007). Thus, it has been shown that loop C contributes to the molecular recognition of the agonist by moving into a capped position and locking the agonist in place (Celie et al., 2004; Gao et al., 2005, 2006; Olsen et al., 2014). Movement of loop C is also involved in the initial steps that lead from binding to gating of the receptor (Sine and Engel, 2006).

The observation that the W55T mutation in loop D of the complementary component of the *α*9 (but not the *α*10) receptor subunit impaired [^3^H]-*α*-BTX binding most likely suggests a disrupted agonist binding site, and therefore that *α*9 contributes to the complementary component of the ligand binding site. In a crystal structure of *α*-BTX bound to a pentameric *α*7/AChBP chimera, while Y190 in loop C is the main contributor to the high-affinity toxin interaction through *π*-cation and hydrogen bond interactions (Huang et al., 2013; Sine et al., 2013) W55 contacts F32 of the toxin and its mutation produces mild but significant reduction of *α*-BTX binding affinity (Sine et al., 2013). The notion that *α*9 contributes to the complementary face of the binding site is further supported by the docking analysis, where in rat receptors the most frequent conformations with ACh in the correct orientation at the binding site were observed at the interface in which *α*10 contributes to the principal (+) and *α*9 to the complementary face (−) interface [*α*10(+)*α*9(−)]. Expression studies of mutant W55T receptors also indicate that *α*9 complementary components contribute to receptor function. The increase in ACh apparent affinity of *α*9W55T*α*10 might also result from reduced gating kinetics. In this regard, mutations in this residue in the muscle receptor affect channel gating due to a reduction in the channel opening rate constant (Akk, 2002).

The fact that the *α*9*χα*10*χ*W55T mutation bound [^3^H]*α*-BTX (and this was displaced by ACh), together with the finding that the *α*9*α*10W55T mutant receptors had similar ACh apparent affinity and macroscopic currents to wild-type receptors, indicates that either *α*10 does not contribute to the complementary face of the binding pocket or that *α*10 might inefficiently provide the (−) face since W55 in loop D cannot make the proper cation-*π* interactions with ACh. The latter is rather unexpected, since W55 is a key contributor of the (−) face to ACh binding in all nAChRs (Karlin, 2002; Olsen et al., 2014). However, it can explain the observation that *α*10 contributes to the complementary face in the presence of disrupted *α*9(−) faces, as observed in functional studies with *α*9W55T*α*10 receptors. Therefore, one could conclude that in rat heteromeric *α*9*α*10 receptors the contribution of *α*10 to the complementary component is nonequivalent to that of *α*9 since it does not involve equally W55, a key residue for ACh binding and gating. This resembles what has been described for the *Torpedo* and muscle embryonic nAChRs, where the contribution of the *γ* and *δ* subunits to the (−) face is nonequivalent (Sine and Claudio, 1991; Martin et al., 1996; Xie and Cohen, 2001). Overall the functional results are in line with the in silico modeling, which showed a significant reduction in the frequency of conformations with ACh docked in the correct orientation with the rat *α*10 subunit placed in the complementary face, *α*9(+)*α*10(−) or *α*10(+)*α*10(−).

The observation that in chicken receptors the introduction of the W55T mutation in either *α*9 or *α*10 produced similar shifts in the ACh apparent affinity of resultant heteromeric receptors indicates that both *α*9 and *α*10 can equally contribute to the (−) face of the binding pocket. This is supported by the observation that, contrary to that observed for rat receptors, in chicken molecular docking studies indicate that the frequency of ACh bound in the correct orientation is similar for either *α*9(+)*α*10(−) or *α*10(+)*α*9(−) interfaces. This might explain that, in contrast to that observed for rat subunits (Elgoyhen et al., 2001; Sgard et al., 2002), chicken homomeric *α*10 receptors are functional when expressed in *Xenopus laevis* oocytes (Lipovsek et al., 2014).

The asymmetry between rat and chicken receptors most likely derives from the acquisition of nonsynonymous substitutions in the complementary face of mammalian *α*10 subunits (Franchini and Elgoyhen, 2006). R117 present in mammalian *α*10 subunits, but replaced by a noncharged methionine or threonine in nonmammalian *α*10 subunits and threonine in vertebrate *α*9 subunits (Fig. 8), might account for the fact that W55 does not equivalently contribute to receptor function when comparing rat *α*10 to rat *α*9, chicken *α*9, and chicken *α*10 subunits. Its presence might result in a positively charged environment that would perturb the access of the quaternary ammonium of ACh to the binding pocket. This resembles what has been recently described in the crystal structure of the *α*4*β*2 nAChR, where three hydrophobic groups on the (−) side of the *β*2 subunit are replaced by polar side chains on the (−) side of the *α*4 subunit. It has been suggested that this difference in chemical environment may affect agonist binding to *α*4–*α*4 interfaces in the (*α*4)_3_(*β*2)_2_ stoichiometry, being a polar environment less favorable for agonist binding (Morales-Perez et al., 2016). Understanding the underlying mechanisms accounting for the perturbation produced by R117 in the (−) face of the rat *α*10 subunit would require further experiments, including determination of the crystal structure of the *α*9*α*10 receptor bound to ACh. However, by double-mutant cycle analysis we have been able to show that W55 and R117 are coupled to each other in their contribution to nAChR function. Thus, the mutation at one site has structural or energetic impact at a second site. Typically, a value of Ω that deviates significantly from 1 is interpreted as a direct interaction between residues, such as that provided by a hydrogen bond or a salt bridge. However, the molecular structure of the *α*9*α*10 nAChR (Fig. 7) shows that W55 and R117 are not in close apposition and appear separated by about 10 Å, thus suggesting that the coupling does not arise from a direct interaction. The occurrence of long-range functional coupling between residues in which a direct interaction is precluded has been described in the mouse muscle nAChR (Gleitsman et al., 2009).

In conclusion, we have demonstrated that whereas both *α*9 and *α*10 contribute to the principal component of *α*9*α*10 nAChRs their contribution to the complementary face of the binding pocket in rat *α*9*α*10 nAChRs is nonequivalent. This results from the adaptive evolutionary amino acid changes acquired by mammalian *α*10, which rendered a divergent branch within the clade of vertebrate *α*10 subunits (Lipovsek et al., 2012).

## Abbreviations

5-HT3A: serotonin type 3A
*α*-BTX: *α*-bungarotoxin
ACh: acetylcholine
ANOVA: analysis of variance
BBE: best binding energy
CC/SS: double cysteine to serine
nAChR: nicotinic acetylcholine receptor
